# Supplementary material for: Characterizing Cancer Drug Response and Biological Correlates: A Geometric Network Approach
Source: Sci Rep. 2018 Apr 23;8:6402. doi: 10.1038/s41598-018-24679-3 (PMC5913269; doi:10.1038/s41598-018-24679-3)
Supplement: Supplementary file 1 — Supplementary Information [file 41598_2018_24679_MOESM1_ESM.pdf]

Characterizing Cancer Drug Response and Biological Correlates: A Geometric Network Approach

Maryam Pouryahya[1,2], Jung Hun Oh[2], James C. Mathews[2], Joseph O. Deasy[2], and Allen R. Tannenbaum[1,3,*]

[1]Department of Applied Mathematics & Statistics, Stony Brook University, Stony Brook, 11794, USA.
[2]Department of Medical Physics, Memorial Sloan Kettering Cancer Center, New York, 10064, USA.
[3]Department of Computer Science, Stony Brook University, Stony Brook, 11794, USA.
[*]allen.tannenbaum@stonybrook.edu

Scientific Reports Supplementary Information

Table S1 presents genes with the highest absolute value of Ricci curvature. We expect these genes to have significant contribution to the network's robustness. Notably, the top two genes are TP53 (tumor protein 53) and YWHAG (see Fig. S1). TP53, also known as p53, is a tumor suppressor gene known as the "guardian of the genome" given the essential role it plays in genetic stability and prevention of cancer formation[1,2]. Mutations in this gene play a role in all stages of malignant transformation including tumor initiation, promotion, aggressiveness, and metastasis[3]. Mutations of this gene are present in more than 50% of human cancers including leukemia, breast cancer, CNS cancers, and lung cancers, among many others, making it the most common genetic event in human cancer[4–9].

The YWHAG gene encodes the 14-3-3 protein gamma, a member of the 14-3-3 family proteins which are involved in many biological processes including signal transduction regulation, cell cycle progression, apoptosis, cell adhesion and migration[10,11]. Notably, increased expression of 14-3-3 family proteins, including protein gamma, have been observed in a number of human cancers including lung and colorectal cancers, among others, suggesting a potential role as tumor oncogenes[12,13]. Furthermore, there is evidence that loss of p53 function may result in up-regulation of 14-3-3γ in lung cancer[11].

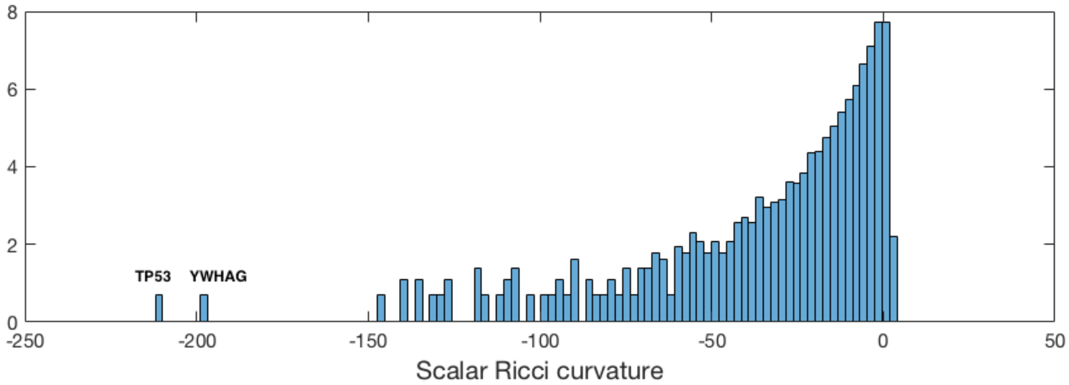

Supplementary Fig. S 1. The histogram of scalar Ricci curvature of 8240 genes. Most of the genes have negative scalar Ricci curvature (75%). TP53 and YWHAG have notably low Ricci curvatures. We normalized the height of the bars with the logarithm function for better visualization.

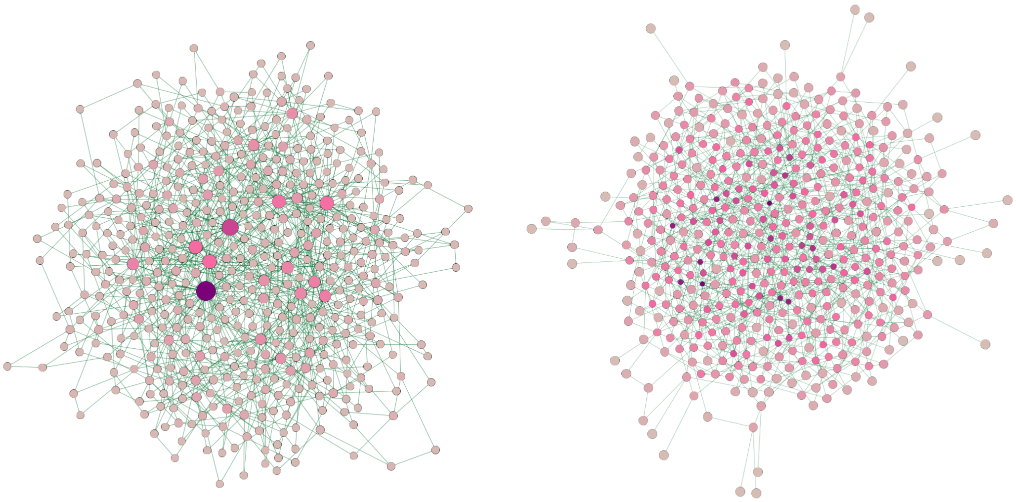

(a) (b)

Supplementary Fig. S 2. (a) Scale-free network with average Ricci curvature of -0.78. (b) Erdös-Rényi network with average Ricci curvature of -0.96. Both networks consist of 500 nodes and ~ 2000 edges. The size of the plot shows the degree of the node and the color of the node corresponds to Ricci curvature. As the Ricci curvature becomes highly negative the color of the node is darker red. As discussed by A.-L. Barabási (Network Science (2014)), the scale-free property strongly correlates with the network's robustness to failure due to having hubs within the network. Here, we see that it also possesses a higher average Ricci curvature.

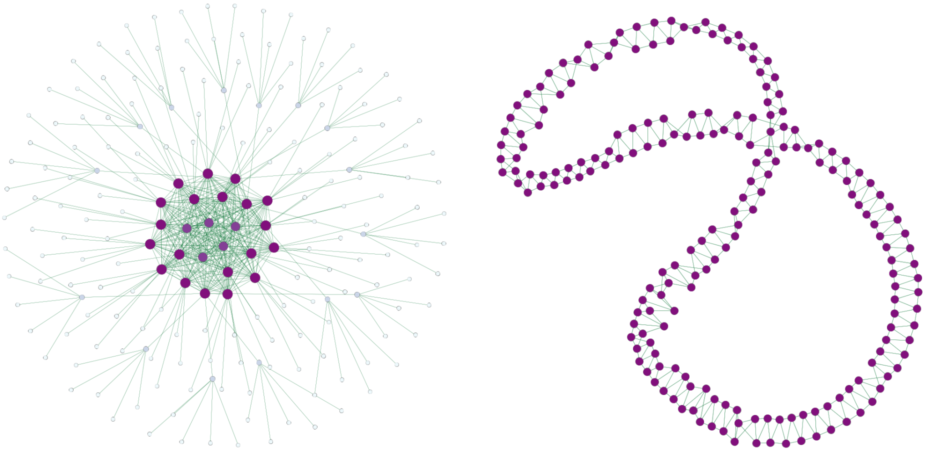

(a) (b)

Supplementary Fig. S 3. (a) Star-shaped network with average Ricci curvature of 0.18. (b) Ring-shaped network with average Ricci curvature of 0.03. Both networks consist of 200 nodes and ~ 800 edges. The star-shaped network has a fully connected core and we expect it to be more robust to random perturbation than the ring-shaped network. Here it has been shown that it also possesses a higher average Ricci curvature.

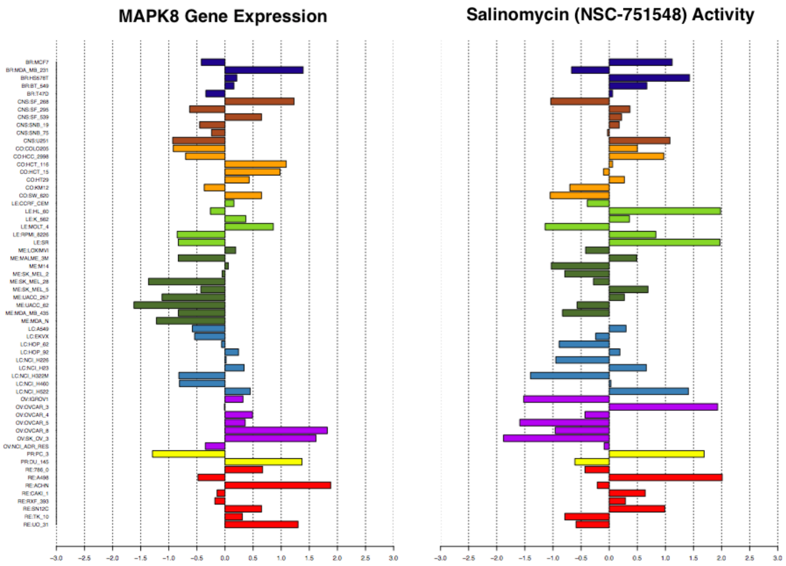

Supplementary Fig. S 4. The Spearman correlation along cell lines between Salinomycin's GI50 activity and MAPK8 expression is significant ($Rs = -0.38$, p-value=0.004). Therefore, MAPK8 is chosen as a significant gene for Salinomycin. The correlation matrix (D) shown in Figure 2 consists of such correlations for every drug/gene pair.

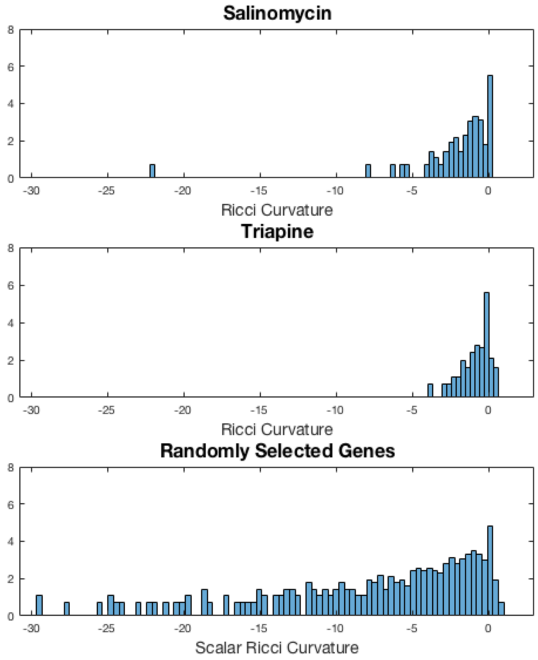

Supplementary Fig. S 5. Histogram of Ricci curvature distribution of genes. The first row corresponds to the first ranked drug, Salinomycin (with ~ 400 significant genes), and the middle row to one of the last ranked drugs, Triapine (ranked 104), with a similar number of significant genes. The bottom row is the Ricci curvature distribution of 400 randomly chosen genes (from 8240 genes). Salinomycin has more nodes with highly negative scalar curvature than Triapine. Whereas, the randomly selected genes have more uniform distribution of Ricci curvature values. The height of the bars is normalized with the logarithm function for better visualization.

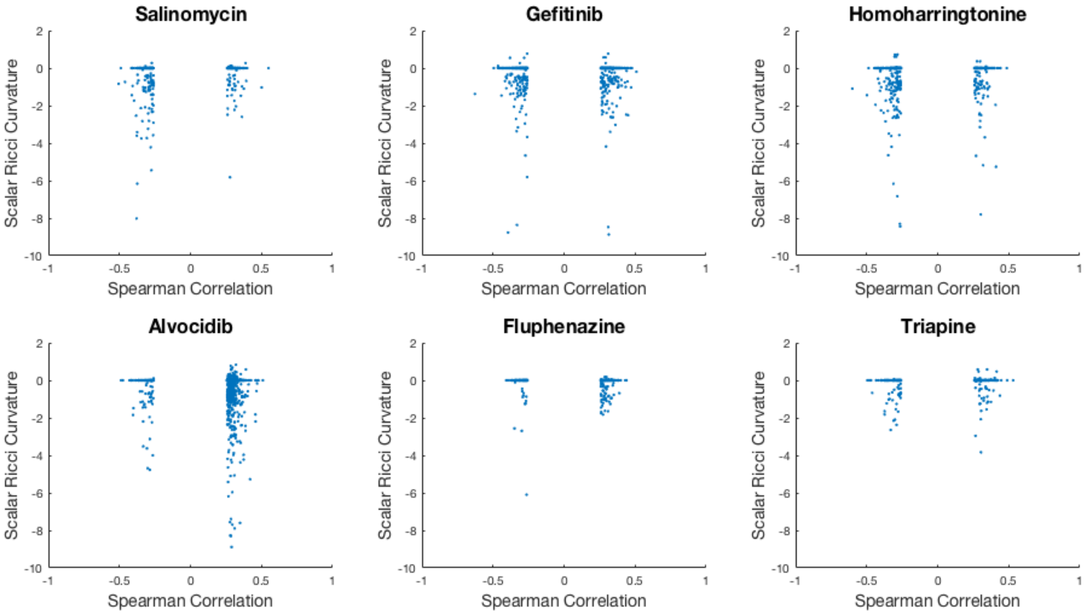

Supplementary Fig. S 6. The calculated Ricci curvature vs. Spearman correlation (between expression and GI50 drug response) of significant genes for the top 3 and bottom 3 ranked drugs. Since these genes are chosen based on the p-value < 0.05, their Spearman correlation is either highly positive or highly negative. The average Ricci curvature is lower in the top ranked drugs (top row), which makes their subnetwork less resistant to the drug. Whereas the bottom row plots are heavier on the right side and have higher average Ricci curvatures.

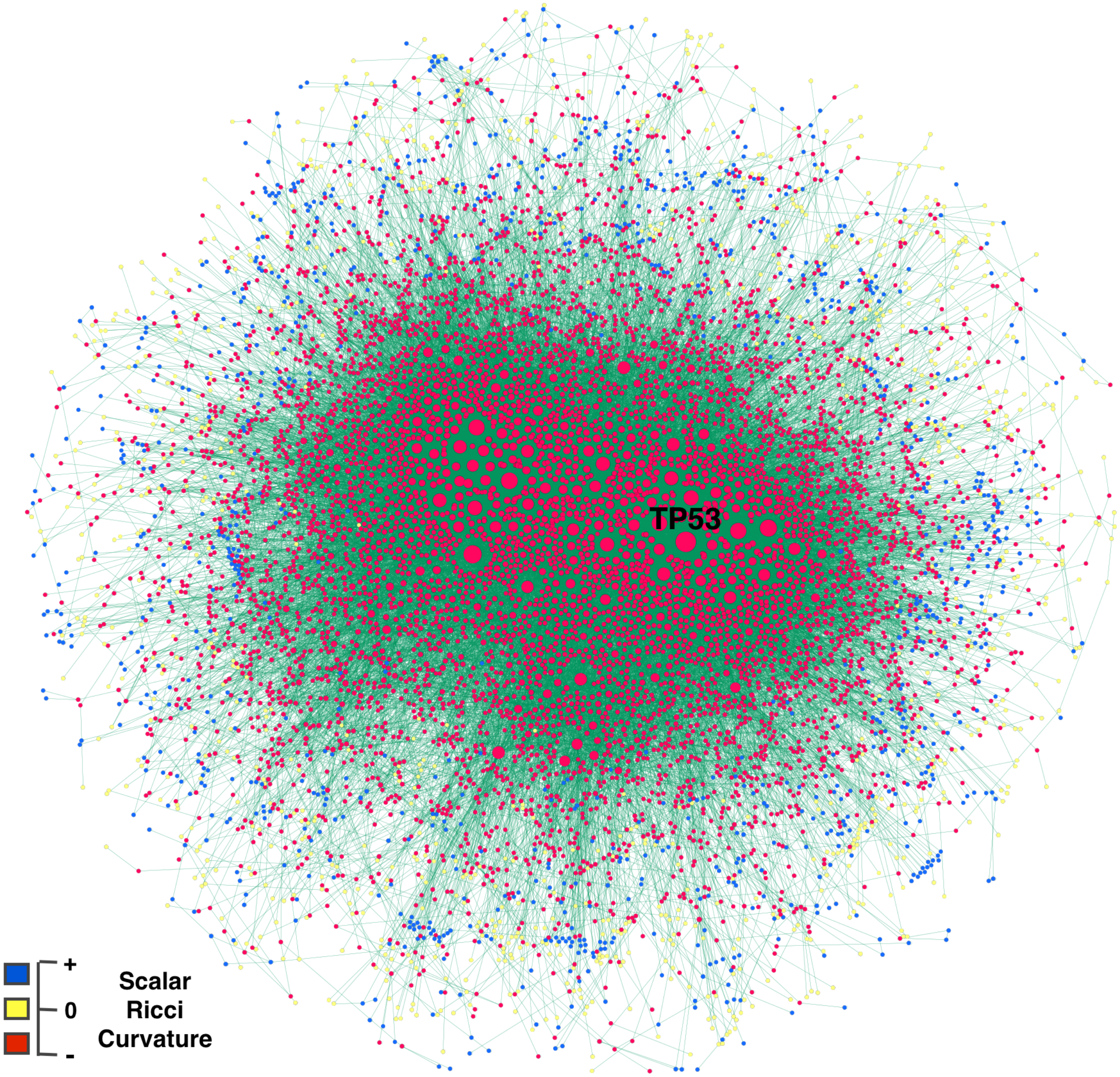

Supplementary Fig. S 7. The pre-treatment network visualization (via Gephi). The network consists of 8240 nodes (genes) and 67235 edges. The sizes of the nodes are proportional to the node-degree and the colors of the nodes correspond to the sign of the scalar Ricci curvature of that node. TP53 has a very high node-degree as well as a distinctively low Ricci curvature.

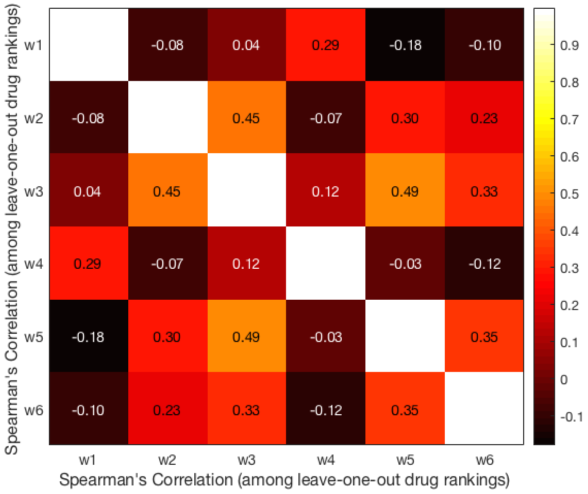

Supplementary Fig. S 8. Color map numbered by excluding one cell line at a time out of 6 cell lines of leukemia. w1: CCRF-CEM (ALL), w2: HL-6-TB (Pro myelocytic), w3: K-562 (CML), w4: MOLT-4 (ALL), w5: RPMI-8226, w6: SR. We see two clusters among the leukemia cell lines, (1,4) and (2,3,5,6). We then compared the drug ranking after adding these two subsets to the other, non-leukemia cell lines. More precisely, we first found the drug ranking with 52 cell lines by excluding all leukemia cell lines. We repeat the pipeline two more times with 54 cell lines (adding (1,4)) and 56 cell lines (adding (2,3,5,6)) and find the drug rankings using these cell lines. After adding (1,4) the Spearman correlation of drug rankings between 54 cell lines and all 58 cell lines is 0.14, yet, by adding (2,3,5,6) the correlation is 0.64. Therefore, the cell lines (1,4) most likely caused the deviation of leukemia from others. Interestingly the two cell lines that caused this deviation belong to the same subtype of leukemia, namely, ALL.

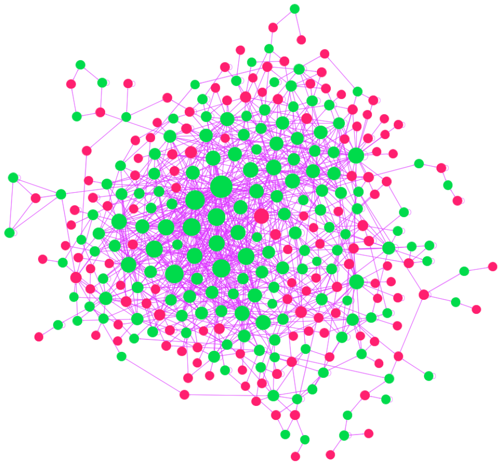

Supplementary Fig. S 9. The subnetwork including the 146 top ranked genes (colored in red). These genes are connected by paths of length two (i.e. two edges), showing that these nodes are not drawn from especially distant parts of the network.

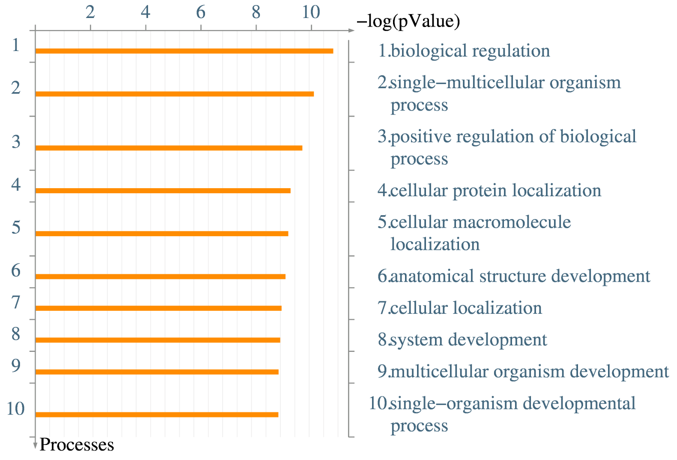

Supplementary Fig. S 10. Top ten biological processes from gene ontology enrichment analysis of the top selected 146 genes considering all cancer types. The corresponding p-values of the top biological processes are also included in the bar plot. The full list of top 50 associated biological processes is provided in Supplementary Table S3.

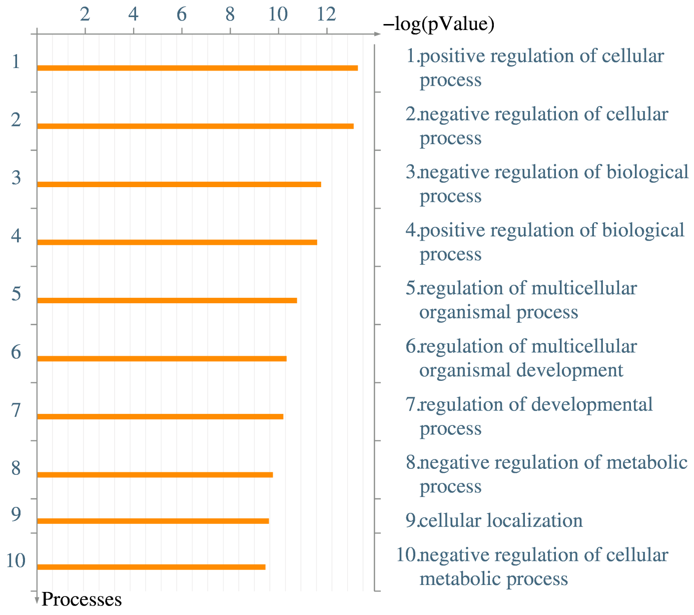

Supplementary Fig. S 11. Top ten biological processes from the gene ontology enrichment analysis of the significant genes correlated to the top ranked drugs of **renal cancer**. Cellular localization is a top ranked biological process for renal cancer as well as all 58 cancer type cell lines.

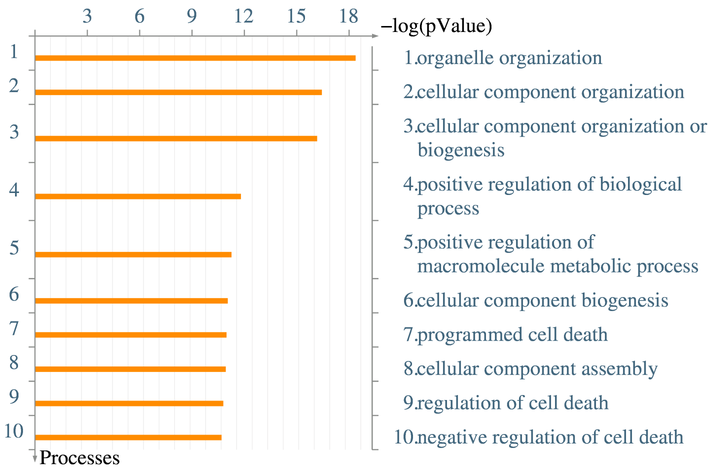

Supplementary Fig. S 12. Top ten biological processes from the gene ontology enrichment analysis of the significant genes correlated to the top ranked drugs of **lung cancer**. Four of the top ranked biological processes are involved with cellular component organization/ assembly.

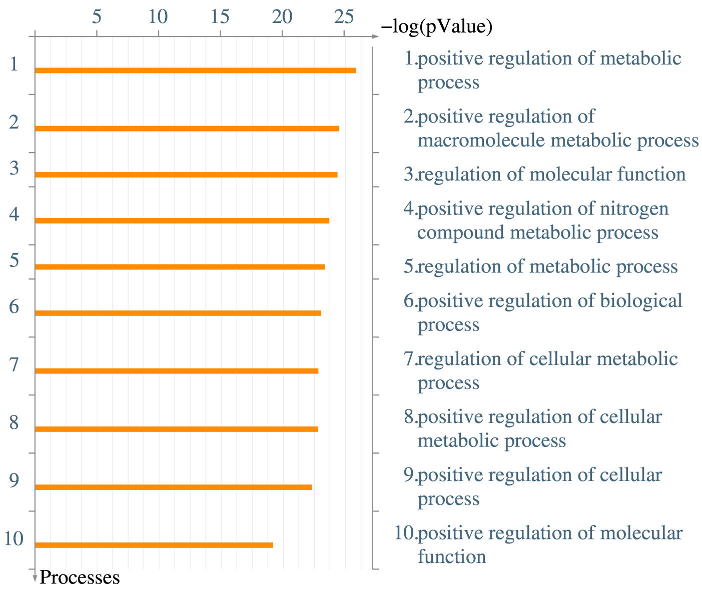

Supplementary Fig. S 13. Top ten biological processes from the gene ontology enrichment analysis of the significant genes correlated to the top ranked drugs of **melanoma**. Most of the top ranked results of melanoma are quite broad terms. Positive regulation of macromolecule metabolic process (ranked 2nd) is also a top biological process for lung cancer.

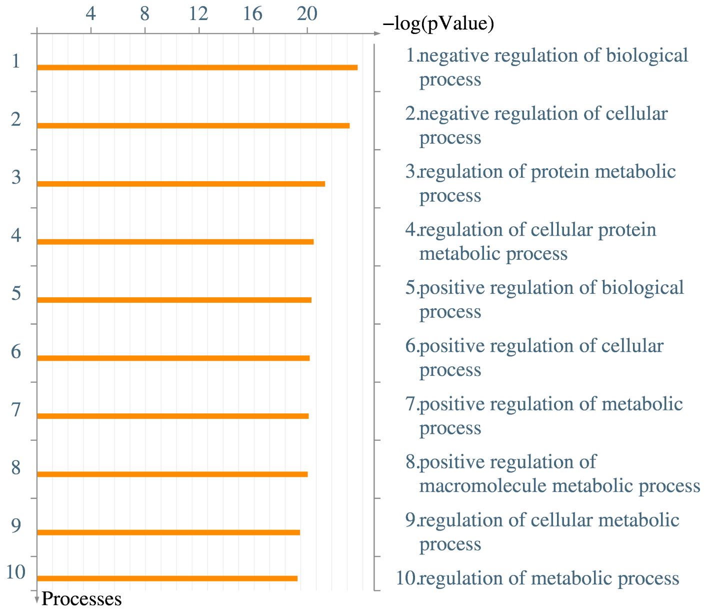

Supplementary Fig. S 14. Top ten biological processes from the gene ontology enrichment analysis of the significant genes correlated to the top ranked drugs of **colon cancer**. Colon cancer has fewer cell lines (7 cell lines) than melanoma (9 cell lines) and lung and renal cancer (8 cell lines). The top ranked biological process (negative regulation of cellular process) in colon cancer is also the second ranked biological process in renal cancer and fifth ranked biological process of all cancer types. However, most of the top ranked results of colon cancer are broad terms.

Gene ranking	Gene name	Gene ranking	Gene name
1	TP53	21	CALM1
2	YWHAG	22	CASP3
3	EP300	23	CCDC85B
4	PRKCA	24	CTNNB11
5	CREBBP	25	VIM
6	CSNK2A1	26	AR
7	UBQLN4	27	CDK1
8	SRC	28	FYN
9	PRKACA	29	RB1
10	SMAD3	30	SMAD9
11	SMAD2	31	YWHAZ
12	GRB2	32	YWHAB
13	TRAF2	33	ACTB
14	ATXN1	34	UBE2I
15	ESR1	35	HDAC1
16	TGFBR1	36	AKT1
17	EWSR1	37	APP
18	EGFR	38	MDFI
19	SMAD4	39	SMAD1
20	MAPK1	40	ACTA1

Supplementary Table S 1. Top 40 genes ranked in ascending order of scalar Ricci curvature in the pre-treatment network.

Ranking	Drug name	Ranking	Drug name	Ranking	Drug name
	Salinomycin	41	Acetalax	80	8-Chloro-adenosine
2	Gefitinib	42	Staurosporine	81	OSI-027
3	Homoharringtonine	43	Eribulin mesilate	82	Chlorambucil
4	Mitomycin	44	Tanespimycin	83	LMP-400
5	Idarubicin	45	Triciribine phosphate	84	Triethylenemelamine
6	Geldanamycin Analog	46	7-Tert-butyldimethylsilyl-10	85	Topotecan
7	Cabozantinib		-hydroxycamptothecin	86	Lapatinib
8	Vinblastine	47	Hydroxyurea	87	Ponatinib
9	PX-316	48	Alvespimycin	88	Belinostat
10	Raloxifene	49	Carmustine	89	Dromostanolone Propionate
11	Pipamperone	50	Nilotinib	90	Dacarbazine
12	Erlotinib	51	Apaziquone	91	Cladribine
13	Fluorouracil	52	Bleomycin	92	Lificguat
14	Imatinib	53	Etoposide	93	Navitoclax
15	Irinotecan	54	Imexon	94	6-Mercaptopurine
16	Doxorubicin	55	RH1	95	Uracil mustard
17	Simvastatin	56	Methotrexate	96	6-Thioguanine
18	Batracylin	57	Estramustine	97	Clofarabine
19	Daunorubicin	58	Cisplatin	98	Pazopanib
20	Azacitidine	59	Teniposide	99	Perifosine
21	Itraconazole	60	Everolimus	100	7-Ethyl-10-hydroxycamptothecin
22	Dasatinib	61	Gemcitabine	101	Calusterone
23	Arsenic trioxide	62	Vemurafenib	102	Allopurinol
24	Ibrutinib	63	Mitoxantrone	103	Fludarabine
25	Tyrothricin	64	Buthionine sulphoximine	104	Triapine
26	Crizotinib	65	Vorinostat	105	Fluphenazine
27	Paclitaxel	66	Dexrazoxane	106	Alvocidib
28	Trametinib	67	Abexinostat		
29	Fenretinide	68	Rapamycin		
30	Tamoxifen	69	Hypothemycin		
31	Floxuridine	70	Okadaic acid		
32	Selumetinib	71	Acrichine		
33	Actinomycin D	72	Sunitinib		
34	Denileukin Diftitox	73	Nandrolone phenpropionate		
35	Cytarabine	74	Thiotepa		
36	Mithramycin	75	Midostaurin		
37	Depsipeptide	76	Carboplatin		
38	Nitrogen mustard	77	Bortezomib		
39	Pipobroman	78	Melphalan		
40	Asparaginase	79	Carfilzomib		

Supplementary Table S 2. Drug ranking of 106 anticancer agents based on the average Ricci curvature of significantly correlated genes.

Ranking	Process	p-value
1	biological regulation	6.053E-08
2	single-multicellular organism process	1.568E-07
3	positive regulation of biological process	2.796E-07
4	cellular protein localization	5.472E-07
5	cellular macromolecule localization	5.472E-07
6	anatomical structure development	5.732E-07
7	cellular localization	6.376E-07
8	system development	6.376E-07
9	multicellular organism development	6.376E-07
10	single-organism developmental process	6.376E-07
11	developmental process	1.092E-06
12	regulation of biological process	1.355E-06
13	tube development	1.660E-06
14	animal organ development	2.022E-06
15	regulation of primary metabolic process	2.817E-06
16	protein localization	2.917E-06
17	positive regulation of cellular process	2.917E-06
18	regulation of nitrogen compound metabolic process	3.296E-06
19	regulation of metabolic process	3.628E-06
20	regulation of macromolecule metabolic process	3.741E-06
21	regulation of cellular metabolic process	3.741E-06
22	macromolecule localization	5.073E-06
23	establishment of localization in cell	6.654E-06
24	organic cyclic compound biosynthetic process	7.872E-06
25	intracellular transport	7.872E-06
26	negative regulation of biological process	7.872E-06
27	maintenance of location	9.820E-06
28	localization	1.401E-05
29	cellular component organization	2.002E-05
30	organelle organization	2.002E-05
31	nucleobase-containing compound biosynthetic process	2.517E-05
32	positive regulation of dendrite morphogenesis	2.517E-05
33	regulation of macromolecule biosynthetic process	2.517E-05
34	multicellular organismal process	2.714E-05
35	regulation of cellular biosynthetic process	2.722E-05
36	regulation of nucleobase-containing compound metabolic process	3.866E-05
37	nervous system development	4.419E-05
38	heterocycle biosynthetic process	4.419E-05
39	aromatic compound biosynthetic process	4.494E-05
40	regulation of cellular process	4.494E-05
41	generation of neurons	4.494E-05
42	cell communication	4.518E-05
43	regulation of biosynthetic process	4.917E-05
44	maintenance of protein location	4.917E-05
45	protein localization to organelle	5.470E-05
46	neurogenesis	5.876E-05
47	negative regulation of cellular process	5.876E-05
48	cellular component organization or biogenesis	6.229E-05
49	cellular developmental process	6.651E-05
50	single-organism cellular process	6.710E-05

Supplementary Table S 3. Top 50 biological processes from the gene ontology enrichment analysis of 146 top selected genes with their corresponding p-values.

Supplementary Table S 4. Top 200 genes selected for the gene ontology enrichment analysis. First column corresponds to all 58 cell line analysis. The second, third, forth and fifth columns are cancer specific results. Columns 1-4 are in the order of genes rankings. The sixth column is the top connected 146 genes which are in alphabetic order.

All	Renal	Lung	Melanoma	Colon	Selected 146
B4GALT3	PRUNE2	BICD2	ANKHD1	TNPO3	ACAT2
BMF	RTN4IP1	KIAA0513	ATP6V0D1	HIVEP1	ADCY6
FAM178B	RTN4R	PEX19	CCT2	TGFBR3	AES
RASSF8	SSBP2	SIGIRR	ETV5	CLCN3	AGFG2
RNF31	SF1	WWC1	EXOC5	CLDN3	AKIRIN2
TEAD2	ADAM19	ZFAND6	GAK	MET	AKR1B1
CCS	CD177	NFE2L2	GMEB2	CD9	ANKHD1
COPS6	ZNF655	PTGES3	ITM2B	FAF1	ANKS1A
DHX15	AASDHPPT	RGS19	MAST2	PTGFRN	AP2A1
PCSK9	CENPF	TFG	MIF4GD	EXOSC8	APPL2
USP53	HINFP	HIST2H2BE	MINK1	ZFP36	ARL6
AGFG2	NR2F2	MIS12	NARF	RSRC1	ARL6IP4
ANKHD1	NR2F6	NACA	NPHP4	GTF2F2	ARL6IP6
FOXP4	NUP133	SMNDC1	PLP2	CCS	ATF5
NCAPG	PLCE1	STX16	PPP1CC	FLNC	ATP2B2
OAZ1	TUBGCP4	TICAM1	PTOV1	SOD1	B4GALT3
RILP	ANXA5	TOP1	SGCB	GNMT	BMF
SAR1A	FKBP4	GPS2	ST3GAL4	PTBP1	BRIP1
ZNF337	GLMN	ABCB8	STIM2	SIPA1L1	C11orf49
NUMBL	RRAD	FAM57A	TLK2	SPARC	CA9
BRIP1	SUPT4H1	ICAM3	TSC22D3	COMMD1	CBS
CREM	SUZ12	SKI	VTI1B	MAPKAPK2	CCDC53
ADCY6	EIF3C	SLC3A2	CALCOCO1	TCHP	CCDC88A
MTSS1	ATP6V0C	TRIM32	ERP27	TOM1L1	CCS
CELSR2	DCP2	USP40	GLRX	USP5	CD3EAP
GMEB2	DDB1	CLINT1	HMGN3	VLDLR	CDK13
SESN2	DPYSL2	GLYCTK	NLE1	TUBGCP4	CDYL
SHMT2	FOXO3	HDHD3	OXSR1	HES6	CELSR2
VPS41	GMEB2	MITF	POU4F1	NEU4	CENPBD1
RANGAP1	KIFAP3	OTX2	SERPINF1	STAM	CLK3
DEXI	KLHL8	RAB11FIP2	TBC1D17	CUL2	COPS6
INVS	PPM1B	SHOC2	TOB2	GLS2	CREM
CDYL	RAPGEF2	STAMBPL1	UQCRFS1	MFN1	CRTC2
FBXW8	SERPINA1	TLN1	IMPDH2	BMP7	CUX1
MAVS	SMAD1	ZMYND11	VPS29	NCBP2	DCC
MIER1	TOX4	TIMM22	VPS35	RMND5B	DHPS
NRSN2	UBTF	SCG5	RPS6KA1	ZP4	DHX15
PDE6D	USP5	ABCD1	MAZ	RAB14	DMPK
PUM1	DNM1L	VAMP4	PDE9A	ATP2B1	DOCK7
SLC12A2	MAP3K10	ADRBK2	REXO1	CASKIN1	DPPA2
SOX4	ATF7IP2	AES	ACAT2	FTH1	DPPA4
TINF2	DARS	BCL7A	DOT1L	IREB2	EIF4EBP1
ZRSR2	FBXO44	KIF11	FBXO30	MT2A	EPS15
SAV1	NEU1	LYPLA1	GNB5	PARVA	EPS15L1
EPS15	NUDT14	MAPK8IP3	GRM3	POLDIP2	ERCC3
CCDC88A	PIGC	PVR	LRP5	SEMA4B	ERH
CBS	POMP	TOMM20	MALT1	SPINK7	EXOC8
EPS15L1	POP4	PKP4	PSMD13	STRADB	FBXW8
NEO1	RSRC1	TNFSF10	PSMD7	CHFR	GJA5
CDK13	RAB9A	HNRNPK	RAD51	PPIE	GMEB2
DOCK7	SAP30BP	CSE1L	RAD54L	RAB13	GOLGB1
JAKMIP1	TM9SF2	CENPF	THAP1	SNF8	GRIK2
ANKS1A	BMP6	ARPC3	WNT1	TFAP2A	HNRNPA2B1
CA9	CACHD1	DMC1	FMNL2	VEGFB	HOOK3
SCP2	DMTF1	RPL6	FKBP2	ADRA2C	HOXA10
KEAP1	DNAJC5	RPL22	OAT	CEBPE	HOXD9
SEC61A1	GBP1	HSPA9	EXOSC2	PRPS1	HSD17B14
PLEKHA5	NEK2	EPN1	ADRBK2	DAAM1	HSF4
SERP1	SIL1	GIGYF1	CAMTA2	HERC5	HSP90B1
DHX38	SNUPN	PPARA	DYRK2	IDO1	IFIT1
GJA5	XRCC1	STRADB	ELP2	PSMB4	JAKMIP1
GSPT2	ARL4A	ZRANB1	GNG11	RBCK1	KCNK9
SYT4	C10orf2	LBR	KDM6A	UBE2M	KIAA2026
SYT6	SLC25A4	POLB	MAP4K2	UBE2Z	KLF10
SYTL3	MARCKS	HMOX1	PPP1R10	ZNF764	LATS1

SS18	FBXO6	IRF8	STAM	ATP5C1	LATS2
SPNS1	RALGPS2	CEP250	TRAF2	METTL1	LHX2
AKR1B1	TXNIP	DNPEP	USP53	CHMP2A	LSR
ARL6IP4	APPBP2	KSR1	NEDD4L	CYB5A	MID2
PRCP	GUSB	NME3	CTNNBIP1	EIF4G1	MIER1
RCAN2	MFSD3	RNF8	FURIN	ISOC2	MTSS1
HOXD9	SLC8A1	SRCAP	ITPR3	POR	NEO1
CENPBD1	MXD1	TFPI2	PLOD3	PPM1B	NOL12
DPPA2	RND3	HDAC7	PPP5C	RHEB	NUMBL
ACAT2	TERF1	RANBP10	SIGMAR1	ZNF510	OAZ1
SF3B3	DLG5	KLHL26	TCEB1	ADCY5	OPHN1
HSD17B14	CCNL2	AKAP10	CCNA1	CASP3	OTX2
DCC	USP8	GCA	SGTA	IFNAR1	PAK3
MID2	A1CF	PSMC1	UBE2I	BAK1	PATZ1
DPPA4	APOBEC1	PSMC2	ADPGK	ADAM33	PDE6D
OTX2	CD22	SRI	CDC20	CLIC1	PEX14
VPS25	CYP11A1	WDR37	FDX1	PPP3CA	PEX6
HOOK3	FBLN1	YBX1	GCC1	MEAF6	PIAS2
PEX14	IGHM	SMG5	GEMIN7	RELA	PIK3AP1
FOXP2	LAMA3	ENAH	HNRNPL	NIPSNAP1	PLEKHA4
PIAS2	STK24	STAT1	MED14	RGS2	PLEKHA5
PAK3	IK	CHORDC1	METTL1	RAB38	PLEKHG2
PRDM1	HIVEP1	MAPRE3	NME3	RHOG	PLIN1
C19orf43	AGR2	PPP5C	PJA1	SDC2	PLIN2
GLRX	CLK2	DCTN1	PRKAR2B	TRA2B	POFUT1
PLEKHA4	ERN1	PTPN3	PSMC3	KHSRP	POLA2
LSR	MAP3K6	SRGAP1	SGOL1	PDCD2	POLE
CCDC53	NDFIP1	CDC25B	TCEB2	PFDN4	PPFIA4
SLC16A1	RAPGEF6	CSNK2A1	TNRC6B	ST13	PRDM1
STIM1	ULK2	HMG20B	VPRBP	DYNLT3	PRDM2
ZNF423	CD46	KIF4A	ARHGAP9	PIAS4	PTPN12
KCNK9	ARL15	NES	RAB11A	SUMO3	PUM1
SRPK2	DKK1	PDCD2	CCDC112	OCRL	RAD54B
ATP2B2	RIPK1	STX6	PPP2R3A	CLU	RANGAP1
ISOC2	CDC23	SPG21	DPM2	TSG101	RASSF8
PPFIA4	ATP1B1	C1QBP	FFAR1	POLA2	RB1
FOXN3	CPSF3	DDB2	GNAI1	RLF	RBM39
HOXA10	ITSN1	EPAS1	LSM3	TERF2IP	RCAN2
PIK3AP1	ERBB2IP	ACTC1	TPX2	IL6	RELL1
PLIN2	CHEK2	HSPH1	XPO5	IPO5	RNF139
RELL1	ASS1	S100A10	TCEA1	SH3KBP1	RNF31
SERPINB1	BAG1	HIP1R	SUPT5H	CAMK2D	RORB
SLC4A2	EIF2S3	RHPN2	AIP	CRIP1	SAR1A
SOX18	MAP4K2	EMB	CIRBP	IL1A	SAV1
TMCO6	PDIA3	RABAC1	RNPS1	KPNA5	SCN8A
ZNF136	PSG9	USF2	ADCY5	LATS1	SEC23IP
RASSF7	SEMA4B	VAMP1	RXRB	SERPINB6	SEC61A1
NOL12	SH3BP2	LIG4	CBFB	TINAG	SERGEF
RWDD3	TPBG	NIN	CHGB	PA2G4	SERP1
DMPK	CCNK	SERTAD2	EGR1	NLRP12	SF3B3
ABHD5	PHPT1	TFDP1	HDAC6	PYCARD	SLC12A2
PLIN1	ADAM15	PSMB6	ARHGEF9	SUMO4	SLC4A1
HNRNPA2B1	DISC1	TRAF7	DLG3	DHCR24	SLC4A2
TXNIP	KCTD5	CENPE	GNAS	CEACAM5	SNW1
LATS1	ATPAF2	RABGGTA	PTPN1	VPS72	SORBS2
SEC23IP	CITED4	RBM4	TRIM32	TERF1	SOX18
AP2A1	DEDD	FAM103A1	SNTA1	EIF2B1	SOX4
STIM2	GABRR2	FAT1	CSNK2A1	MAP3K11	SPNS1
RBM39	ING3	WDR61	POLR2L	PNMA1	SRPK2
MESDC1	KIF1B	TRAF3IP1	HRAS	HMGB1	SS18
RNF139	KIF23	DYRK1B	ETV6	BCOR	STIM1
TMEM139	LRP12	HTRA1	TRAF6	HSD17B4	STIM2
KIF2B	MPP6	POR	NIPBL	APTX	STK25
ERAP1	PGF	CCDC53	WWC1	FBXL12	STK39
RAD54B	PIGK	FAM127A	DAG1	SMARCA4	SYT4
ERCC3	PIGT	CASP7	EIF3J	RNPS1	SYT6
PATZ1	RND2	AFTPH	RSF1	SERPINB9	SYTL3
HSF4	RTN3	ARPC4	USP13	RAP2B	TAF8
RORB	SIRPA	UBN1	PRMT1	NOTCH3	TEAD2
EIF4EBP1	STX5	RANBP3	AKAP11	PPP1R8	TMCO6
KCNQ1	TIMP2	PPIB	RPL6	MDK	TMEM139
CPEB1	TWIST1	TES	PIK3C2B	PRKAB2	TP73
ZC3H7B	VCPIP1	ANXA2	SSSCA1	PRKAG2	TWF1
GOLGB1	LAPTM5	TGS1	PLK2	CCNO	TXNIP

CRTC2
CUX1
TAF8
C11orf49
FAP
SDSL
SCN8A
PRDM2
AES
POFUT1
ERH
TWF1
RB1
AFG3L2
TP73
CD3EAP
STK39
SERGEF
NUP133
APPL2
DHPS
ATF5
POLE
GRIK2
ARL6IP6
LHX2
NPHP3
AKIRIN2
CLK3
DYNC1I2
IFIT1
KLF10
NIPSNAP1
PLEKHG2
PTPN12
RABGAP1
RTN4R
SEC24C
UFD1L
ZBTB6
HSP90B1
OPHN1
ARCN1
POLA2
LATS2
EXOC8
STK25
EXO1
SORBS2
PEX6
KIAA2026
LDHB
ARL6
LSM11
ZNF473
SNW1
CTSF
PHKB
PRKACB
SLC4A1
VDAC3
ROCK1
ZYX
MAX
FAM160A2
HOXB8
RARG
VPS4A
RPS6
C14orf166
NIN
TRIM37
AP2A2
BMP5
COL7A1
DAG1
FZD5
HMG20B
PCMT1
PDGFA
PPP2R5D
SRGN
STAT6
VCL
LRP5
NUCB1
PAK2
RASAL2
ZMIZ1
NAT9
WDR77
ATG12
EHD1
SAE1
HADHB
ZMIZ2
UBE2E3
AP4E1
EGLN3
EIF3J
ACVR1B
STAG2
CDK5RAP2
GADD45A
OLFM2
RPE
TBC1D4
TSN
STAG1
PCBD1
AP2A1
PPARD
CIR1
HMG20A
GSTP1
ATP5B
C1QTNF1
CDCP1
NDUFS7
PCDH1
ROCK2
SNPH
DYNC1I2
RBM14
DNAJB9
SMARCC1
MED31
ABCD3
COL6A2
AIP
TRIP13
PIM1
CSF2
NAPA
FAM101B
GABPB1
TNFRSF10B
DST
FTL
MUC4
PRKCE
UROS
RIT1
CPSF3L
RAB5A
SH3BP4
SMARCD1
KITLG
KIAA1549
KRT81
PLEKHM1
HRK
KRT15
RAB1A
TRIAP1
PTPN6
ARHGEF12
CNTNAP4
DAB2IP
FGFR2
LIME1
MAD2L2
MED4
NINL
POLR1A
POLR1E
REV3L
SIX1
TBCD
VPS36
MAST2
ORAI2
SPRY2
LGALS13
EWSR1
SERPINA1
MED6
CHD9
GNA11
NRIP1
RAN
EIF3E
SDC1
BLMH
CASP2
COIL
FTL
HOXA1
HSPA13
PLCD1
TCF7L2
RABAC1
TAF10
NKRF
C10orf2
CHD4
DKK1
GTF2A2
INVS
NDOR1
NECAP1
RAB18
RPS18
SORBS3
STAG1
TNPO1
TRIP12
WDR37
COPG2
MARK4
CFDP1
FANCD2
NCSTN
PABPN1
RAB22A
RAB5C
TNKS2
E2F5
ACD
PLA2G4A
BNIP2
NDFIP1
FUNDC2
SFN
ACACA
RRAS2
POLH
POLDIP2
RPS15A
GABARAPL2
PCBP1
MAPK12
AURKA
CAPNS1
DRG1
GCH1
NDEL1
PSMD1
RLF
ADA
VARS
TAF4
PRPF3
VPS28
ADAM10
GTF2H5
NGEF
STRBP
GNAZ
KAT2B
SH3GL2
NBN
PDK2
APP
APOBEC1
IDH1
RAB2A
SENP6
PPARA
VAMP1
HIC2
SLC1A1
GADD45B
ZBTB43
XRCC4
CTTN
APBA2
NMT2
SIRT1
ABI2
BACE1
IGF2BP2
MAP2K4
BAZ1B
MYO1C
HOOK1
VPS41
MAFG
CYFIP2
CBS
GRIPAP1
KCNH2
MID1
MID1IP1
UBE2D3
MED17
ITSN1
TPM1
TRAF3IP2
ARID2
PSMC4
PRPF4
RAP1B
MINK1
RNMT
SMARCE1
PCBP1
TP53BP2
KPNA2
SMURF2
UROS
AFF4
ANKRD11
HDHD3
PLAC8
VDAC3
VPS41
ZBTB6
ZC3H7B
ZNF337
ZNF423
ZRSR2

References

1. Lane, D. P. Cancer. p53, guardian of the genome. *Nature.* **358(6381)**, 15-16 (1992).
2. Surget, S., Khoury, M. P. & Bourdon J. C. Uncovering the role of p53 splice variants in human malignancy: a clinical perspective. *Onco. Targets Ther.* **7**, 57-68 (2013).
3. Rivlin, N., Brosh, R., Oren, M. & Rotter, V. Mutations in the p53 Tumor Suppressor Gene: Important Milestones at the Various Steps of Tumorigenesis. *Genes Cancer.* **2(4)**, 466-474 (2011).
4. Levine, A. J. & Oren, M. The first 30 years of p53: growing ever more complex. *Nat. Rev. Cancer.* **9(10)**, 749-758 (2009).
5. Freed-Pastor, W. A., Prives, C. Mutant p53: one name, many proteins. *Genes Dev.* **26(12)**,1268-1286 (2012).
6. Zhao, Z., *et al.* p53 loss promotes acute myeloid leukemia by enabling aberrant self-renewal. *Genes Dev.* **24(13)**, 1389-1402 (2010).
7. Gasco, M., Shami, S. & Crook, T. The p53 pathway in breast cancer. *Breast Cancer Res.* **4(2)**, 70-76 (2002).
8. Jin, Y., Xiao, W., Song, T., Feng, G. & Dai, Z. Expression and Prognostic Significance of p53 in Glioma Patients: A Meta-analysis. *Neurochem. Res.* **41(7)**, 1723-1731 (2016).
9. Ahrendt, S. A., *et al.* p53 mutations and survival in stage I non-small-cell lung cancer: results of a prospective study. *J. Natl. Cancer Inst.* **95(13)**, 961-970 (2003).
10. Horie, M., Suzuki, M., Takahashi, E. & Tanigami A. Cloning, expression, and chromosomal mapping of the human 14-3-3gamma gene (YWHAG) to 7q11.23. *Genomics.* **60(2)**, 241-243 (1999).
11. Chen, D. Y., Dai, D. F., Hua, Y., Qi, W. Q. p53 suppresses 14-3-3γ by stimulating proteasome-mediated 14-3-3γ protein degradation. *Int. J. Oncol.* **46(2)**, 818-824 (2015).
12. Qi, W., Liu, X., Qiao, D. & Martinez, J. D. Isoform-specific expression of 14-3-3 proteins in human lung cancer tissues. *Int. J. Cancer.* **113(3)**, 359-363 (2005).
13. Radhakrishnan, V. M. & Martinez J. D. 14-3-3gamma induces oncogenic transformation by stimulating MAP kinase and PI3K signaling. *PLOS One.* **5(7)**, e11433 (2015).
